# Clinical significance of serum MBD3 detection in girls with central precocious puberty

**DOI:** 10.1515/med-2022-0614

**Published:** 2023-07-07

**Authors:** Lili Zhou, Xiao Jia, Xiangzheng Yang

**Affiliations:** Department of Pediatrics, Beijing University of Chinese Medicine Shenzhen Hospital (Longgang), No. 1 Dayun Road, Shenzhen, 518000, China; Department of Orthopaedics, Gansu Provincial Hospital of TCM, Lanzhou, 730050, China; Department of Pediatrics, Beijing University of Chinese Medicine Shenzhen Hospital (Longgang), Shenzhen, 518000, China

**Keywords:** central precocious puberty, girls, MBD3, basal luteinizing hormone, peak luteinizing hormone, basal follicle-stimulating hormone, peak follicle-stimulating hormone, GnRH

## Abstract

Diagnosis of central precocious puberty (CPP) in girls remains a huge challenge. The current study was to measure the serum expression of methyl-DNA bind protein 3 (MBD3) in CPP girls and assess its diagnostic efficacy. To begin with, we enrolled 109 CPP girls and 74 healthy pre-puberty girls. Then, MBD3 expression in their serum samples was measured via reverse transcription-quantitative polymerase chain reaction, and its diagnostic efficacy on CPP was assessed via the receiver operating characteristic (ROC) curve, followed by correlation analysis between serum MBD3 and patient age, gender, bone age, weight, height, body mass index, basal luteinizing hormone (LH), peak LH, basal follicle-stimulating hormone (FSH), peak FSH, and ovarian size using bivariate correlations method. Finally, independent predictors of MBD3 expression were confirmed using multivariate linear regression analysis. MBD3 was highly expressed in sera of CPP patients. The area under the ROC curve of MBD3 diagnosing CCP was 0.9309, with 1.475 cut-off value (92.66% sensitivity and 86.49% specificity). MBD3 expression positively correlated with basal LH, peak LH, basal FSH, and ovarian size, among which basal LH was considered the strongest independent predictor of MBD3, followed by basal FSH and peak LH. In summary, serum MBD3 could act as a biomarker in aiding CPP diagnosis.

## 1 Introduction

Precocious puberty, in its essence, represents the emergence of secondary sexual characteristics in girls under 8 years old and boys under 9 years old [1]. The advancement of pubertal timing might be the result of abnormal interactions among hormonal, metabolic, genetic, ethnic, and environmental factors [2]. In clinical terms, precocious puberty is classified into complete precocious puberty, central precocious puberty (CPP), and incomplete precocious puberty [3]. CPP refers to physiological pubertal development at an inappropriate age attributed to the prematurely activated hypothalamic–pituitary–gonad (HPG) axis and featured by increased production and release of gonadotropin-releasing hormone (GnRH) [4]. Compared to boys, girls are more susceptible to CCP, and risks of short and slight stature, obesity, consequent psychosocial distress, and other diseases including cardiovascular disease and diabetes are high when girls with CCP enter adulthood [5,6]. Therefore, girl-centered research should be given higher priority in the study concerning CPP diagnosis and treatment.

The widely used diagnostic methods for CPP consist of stimulation with GnRH or GnRH analogs (GnRHa), estimation of luteinizing hormone/follicle-stimulating hormone (LH/FSH) ratio, and pelvic ultrasound [7]. Pelvic ultrasound is the additional method used when the results of the GnRH stimulation test, often expensive and time-consuming, are ambiguous [6,8]. Combined with the above-mentioned reality, more additional tools are in urgent need to achieve an accurate and rapid diagnosis of CPP.

Researchers have identified several serum biomarkers that possess great significance in CPP diagnosis, such as neurokinin B and kisspeptin [9], Makorin ring finger protein 3 (MKRN3) [10], and anti-Müllerian hormone and inhibin B [11]. Methyl-DNA bind protein 3 (MBD3), a member of methyl binding proteins, is implicated in the manipulation of puberty initiation in mammals [12]. MBD3 poses an influence on the puberty of mammals and thus affects their sexual competence [13]. Nevertheless, the single function and significance of serum MBD3 in CPP clinical detection have not been elucidated up to date. The current study intended to figure out the clinical significance of serum MBD3 in CPP girls, and thus provide a new perspective for the diagnosis and management of CPP.

## 2 Materials and methods

### 2.1 Ethics statement

The study protocols complied with the Declaration of Helsinki and were ratified by the Scientific Ethics Committee of Beijing University of Chinese Medicine Shenzhen Hospital (Longgang). Informed consent was obtained from each participant.

### 2.2 Study subjects

Participants included girls with CPP (*N* = 109) who were recruited from the pediatric endocrine clinic at Beijing University of Chinese Medicine Shenzhen Hospital (Longgang) and healthy pre-puberty girls (*N* = 74, control group) who were admitted to Beijing University of Chinese Medicine Shenzhen Hospital (Longgang) for physical examination from June 2018 to June 2021. The age of all participants ranged from 6 to 9 years.

Inclusion criteria were as follows: (1) patients showed Tanner breast stage scores ≥2 based on standardized Tanner breast stage assessment before the age of 8 years, (2) bone age (BA) of patients assessed by a single observer should be at least 1 year ahead of their actual age following the Greulich and Pyle method, and (3) peak LH was higher than the critical value of 5 IU/L in the GnRH test before the age of 9 years.

Further laboratory and imaging studies were carried out following clinical judgment to exclude other forms of precocious puberty. Exclusion criteria were as follows: (1) patients with clear etiology such as brain tumor or cranial irradiation and (2) patients with a history of GnRH agonist treatment.

### 2.3 Collection of clinical baseline characteristics

The body mass index (BMI) of each participant was calculated by the equation: BMI = body weight/height^2^ (kg/m^2^). The stage of puberty was determined in accordance with the Marshall and Tanner criteria [14]. BA was estimated using the Greulich and Pyle method [15]. The ovarian and uterine ultrasonic examination was conducted by an experienced radiologist.

### 2.4 Blood sample collection

Participants fasted for 12 h overnight and 8 mL blood sample was collected in the morning and immediately centrifuged. Serum samples were transferred to Eppendorf tubes and conserved at −80°C. Morning basal serum levels of LH and FSH in all patients were measured using chemiluminescent immunoassay kits (Immulite 2000, Siemens, Eschborn, Germany) [16].

### 2.5 GnRH stimulation test

GnRH stimulation test was conducted on fasted CPP patients using gonadorelin acetate (LHRH Ferring^®^, Ferring Pharmaceuticals Inc., Tarrytown, NY, USA) between 8 and 8:30 a.m. [17]. Patients were intravenously administered with GnRH (0.1 mg/m^2^) and samples were collected at 20, 40, 60, and 90 min after administration to measure FSH and LH. Peak LH >5 IU/L was considered to be indicative of puberty [18].

### 2.6 Reverse transcription-quantitative polymerase chain reaction (RT-qPCR)

Total RNA was extracted from serum samples utilizing TRIzol kits (Invitrogen life, Carlsbad, CA, USA), followed by determination of RNA quantity and purity using a nano-drop spectrometer (Thermo Fisher Scientific, Waltham, MA, USA). Optical density (OD) of RNA at 260/280 nm was detected, and RNA samples with the OD value of 1.8–2.2 at 260/280 nm were used for cDNA synthesis. Subsequently, 2 μg RNA was subjected to reverse transcription using SuperScript^TM^ III Reverse Transcriptase (Invitrogen Life Technologies) as per the manufacturer’s protocols. Afterward, RT-qPCR was implemented to quantify MBD3 expression utilizing Gene Amp PCR System 9700 (ABI, Foster City, CA, USA) and 2X PCR Master mix (KANGCHENG, Shanghai, China). The 2^−ΔΔCt^ method was adopted to calculate the relative expression of MBD3 with GAPDH as an internal reference [19]. The primer sequences were as follows: MBD3, F: 5′-ATGCTGATGAGCAAGATGAAC-3′, R: 5′-GGCTCTTGTTCATCTTGCTCA-3′; GAPDH, F: 5′-CCCACTCCTCCACCTTTGAC-3′, R: 5′-CATACCAGGAAATGAGCTTGACAA-3′.

### 2.7 Statistical analysis

Data analysis and drafting were performed utilizing SPSS 21 (IBM Corp., Armonk, NY, USA) and Graphpad Prism 8 software. Normal distribution of data was confirmed by Shapiro–Wilk test, and the value beyond 0.05 was perceived as normal. Values of normally distributed continuous variables were expressed as mean ± standard deviation (SD), whereas values of abnormally distributed variables were expressed as the median and interquartile range. Normally distributed continuous variables were compared using Student’s *t*-test and one-way analysis of variance, and abnormally distributed ones were compared using Mann–Whitney and Kruskal–Wallis non-parametric test. The sensitivity and specificity of MBD3 were estimated by the critical value identified in the receiver operating characteristic (ROC) curve, and the correlation among variables was analyzed using bivariate analysis. Significant predictors affecting MBD3 expression were confirmed by multivariate linear regression analysis. The threshold of *P* < 0.05 was regarded as statistically significant.

## 3 Results

### 3.1 Clinical baseline information

Altogether 109 CPP patients (average age: 7.19 ± 0.79 years) and 74 healthy participants (average age: 7.18 ± 0.72 years) were enrolled in the present study. The ratio of CPP patients at the Tanner stage of II/III was 84/25. The clinical baseline characteristics of participants are illustrated in Table 1. Compared with the control group, the CPP group exhibited great differences in BA, BA advancement, height, weight, basal LH, basal FSH, and the size of left and right ovaries (all *P* < 0.05), and no distinct difference in mean actual age and BMI.

**Table 1:** Clinical baseline characteristics

	CPP (*N* = 109)	Normal (*N* = 74)	*P*-value
Age	7.19 ± 0.79	7.18 ± 0.72	0.8926
BA	8.63 ± 0.89	7.75 ± 1.02	<0.001
Age-BA	1.44 ± 0.41	0.57 ± 0.60	<0.001
Height (cm)	130.30 ± 9.69	126.70 ± 8.44	0.0092
Weight (kg)	35.40 ± 5.66	33.47 ± 5.44	0.0225
BMI	21.13 ± 4.23	21.04 ± 3.88	0.8894
Basal LH (IU/L)	1.54 (0.93–2.40)	0.10 (0.08–0.14)	<0.001
Peak LH (IU/L)	13.39 (9.56–17.57)	—	
Basal FSH (IU/L)	3.23 (2.01–4.30)	1.39 (0.79–3.06)	<0.001
Peak FSH (IU/L)	13.39 (9.56–17.57)	—	
Right ovary size (cc)	2.77 ± 1.43	1.60 ± 0.72	<0.001
Left ovary size (cc)	2.86 ± 1.46	1.32 ± 0.64	<0.001

Note: BMI, body mass index; LH, luteinizing hormone; FSH, follicle stimulating hormone; CPP, central precocious puberty. Normally distributed continuous variables were described as mean ± SD; abnormally distributed variables were described as median and interquartile range (IQR).

### 3.2 MBD3 is highly expressed in sera of CPP patients and has higher diagnostic efficacy

**Figure 1:**
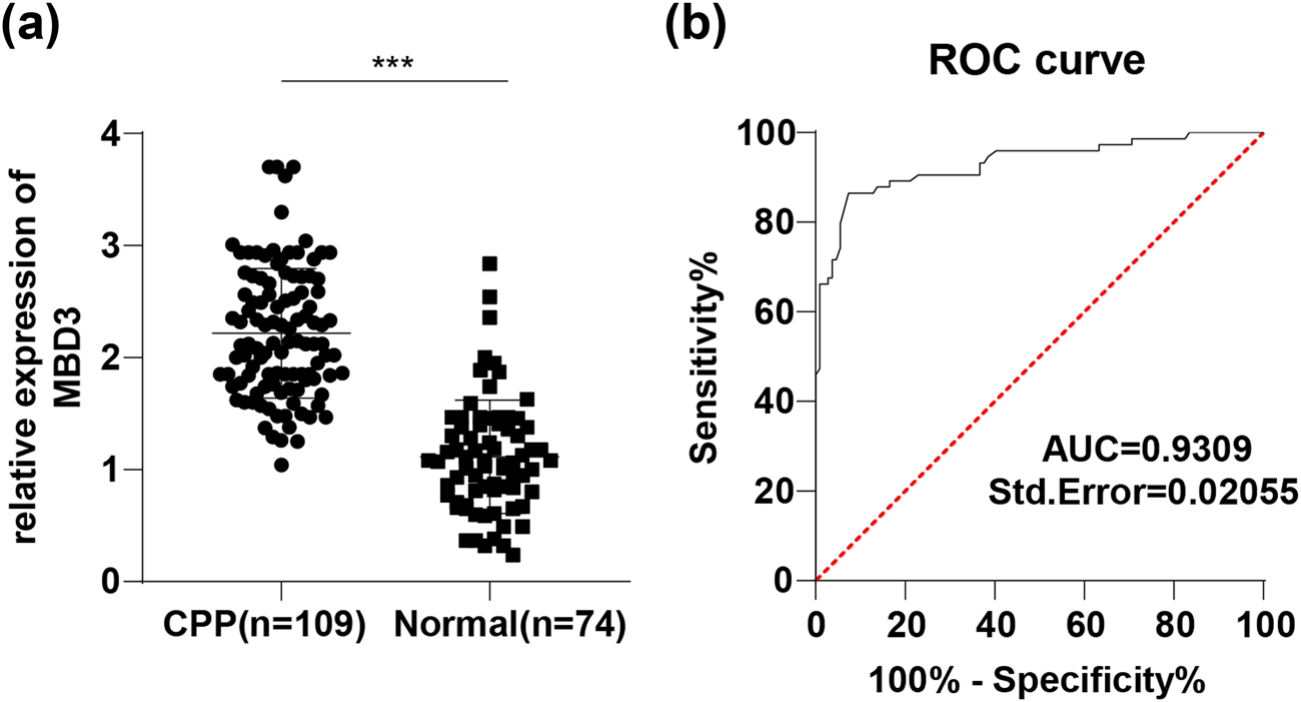
MBD3 is highly expressed in serum of CPP patients and has higher diagnostic efficacy: (a) expression of serum MBD3 measured via RT-qPCR and (b) diagnostic efficacy of MBD3 on CPP patients analyzed via ROC curve. Data in panel (a) were processed utilizing independent sample *t*-test and data in panel (b) were processed by means of ROC analysis. *** *P* < 0.001.

RT-qPCR demonstrated remarkably elevated levels of MBD3 in sera of CPP patients compared with healthy subjects (*P* < 0.001, Figure 1a). To understand whether serum MBD3 expression has diagnostic values on CPP, we draw a ROC curve to distinguish CPP patients from healthy subjects with the expression level of MBD3, which illustrated the area under the curve (AUC) as 0.9309 and cut-off value as 1.475 (92.66% sensitivity and 86.49% specificity) (*P* < 0.0001, Figure 1b). All in all, serum MBD3 expression >1.475 possessed some auxiliary diagnostic values on CPP.

### 3.3 MBD3 shows correlations with clinical baseline characteristics of CPP patients

Current CPP diagnosis depends on GnRH or GnRHa stimulation, LH/FSH ratio, and pelvic ultrasonography [7], yet the features of high expenses and cost-time make it difficult to popularize. MBD3 as an influencing factor of GnRH in mammals might exert auxiliary effects on CPP diagnosis [12]. By analyzing the correlation between serum MBD3 expression in CPP patients and clinical baseline characteristics using bivariate correlation analysis, we disclosed that serum MBD3 expression in CPP patients was positively associated with basal LH (*r* = 0.229, *P* = 0.016), peak LH (*r* = 0.299, *P* = 0.002), basal FSH (*r* = 0.252, *P* = 0.008), and the size of right ovary (*r* = 0.226, *P* = 0.018) and left ovary (*r* = 0.208, *P* = 0.030) (Table 2).

**Table 2:** Correlation between MBD3 and clinical baseline characteristics of CPP patients

		MBD3	
	*N*	*r*	*P*
Age	109	−0.151	0.117
BA	109	−0.165	0.086
Age-BA	109	−0.07	0.467
Height (cm)	109	−0.026	0.788
Weight (kg)	109	−0.047	0.629
BMI	109	−0.025	0.793
Basal LH (IU/L)	109	0.229*	0.016
Peak LH (IU/L)	109	0.299**	0.002
Basal FSH (IU/L)	109	0.252**	0.008
Peak FSH (IU/L)	109	−0.065	0.505
Right ovary size (cc)	109	0.226*	0.018
Left ovary size (cc)	109	0.208*	0.03

Note: MBD3, methyl-DNA bind protein 3; CPP, central precocious puberty; BMI, body mass index; LH, luteinizing hormone; FSH, follicle stimulating hormone. * *P* < 0.05, ** *P* < 0.01.

### 3.4 Basal LH is the strongest predictor of MBD3

Multivariate linear regression analysis was conducted to explore the factors independently affecting MBD3, which revealed peak LH, basal LH, and basal FSH as the independent predictors of MBD3 (Table 3). Among these predictors, basal LH exhibited the strongest correlation with MBD3 expression, followed by basal FSH and peak LH. Collectively, basal LH was regarded as the strongest independent predictor of MBD3.

**Table 3:** Basal LH is the strongest independent predictor of MBD3

Model	Unstandardized coefficients		Standardized	*t*	Sig.
	*B*	Std. Error	Beta		
(Constant)	1.524	0.16		9.532	<0.001
Peak LH	0.013	0.006	0.205	2.182	0.031
Basal LH	0.133	0.05	0.243	2.672	0.009
Basal FSH	0.081	0.034	0.228	2.401	0.018

Note: LH, luteinizing hormone; MBD3, methyl-DNA bind protein 3; FSH, follicle stimulating hormone. Dependent variable: MBD3.

## 4 Discussion

The repercussions of CPP are multi-dimensional, for example, short stature from the physical perspective, psychosocial influences, and long-term risk of potential diseases [20]. Since CPP affects the optimal growth in girls, it is imperative to screen and diagnose patients with CPP as early as possible for chronergy in treatment [21]. This study thereby measured the expression patterns of MBD3 in sera of CPP girls and explored its potential value on CPP diagnosis. It turned out that MBD3 was highly expressed in sera of CPP patients and serum MBD3 could act as a biomarker in aiding CPP diagnosis.

The presence of any two among breast Tanner stage ≥III, basal LH ≥0.2 IU/L, or basal FSH ≥1.6 IU/L implicitly indicates pubertal response [22]. Our study included 109 CPP patients, among who 84 were at the Tanner II stage and 25 were at the Tanner III stage. All enrolled CPP patients showed basal LH above 0.2 IU/L and basal FSH above 1.6 IU/L. BA could be used to assess the bone maturation degree and the development potential of individuals in the future [23]. BA advancement is another indicative factor of precocious puberty [24]. LH and FSH secretion is often linked to the emergence of pubertal signs and pubertal growth [25,26]. Evidently, pubertal girls have higher ovarian volumes than pre-pubertal girls [27]. In terms of statistics, CPP patients registered in our study manifested evident differences in BA, BA advancement, height, weight, basal LH, basal FSH, and left/right ovarian size relative to healthy participants.

The early activation of pulsatile GnRH secretion represents the most common mechanism of CPP [4]. MBD3 conveys an activating effect on GnRH1 transcription [13]. Emerging evidence suggests that ubiquitination of MBD3 mediated by MKRN3 plays an inhibitory role in puberty initiation [12]. Our results elicited highly expressed MBD3 in sera of CPP girls. ROC curve presented the impressive diagnostic value of high serum MBD3 expression (>1.475) on CPP, evidenced by the AUC at 0.9309, cut-off value at 1.475, 92.66% sensitivity, and 86.49% specificity. Basal LH values have exhibited effectiveness in establishing diagnosis and monitoring treatment efficiency in CCP girls [28,29]. Peak LH response to GnRH stimulation acts as a dependency factor of CPP diagnosis [30]. There is also evidence that CPP patients have higher basal FSH levels [31]. A former study has confirmed the usefulness of bilateral ovarian enlargement in female isosexual precocious puberty [32]. Our results presented positive associations between serum MBD3 expression and basal LH, peak LH, basal FSH, and ovarian size of CPP patients. Among basal LH, FSH, and LH/FSH ratio, basal LH exhibits the maximal predictability in CPP [29]. As suggested by multivariate linear regression analysis, peak LH, basal LH, and basal FSH were independent predictive factors of MBD3, amongst which basal LH evinced the strongest association with MBD3 expression, and basal FSH and peak LH were next.

To sum up, this article elucidated that serum MBD3 expression could be used as a biomarker to aid the diagnosis of CPP in girls. This was the first time that MBD3 expression was measured in sera of CPP patients. Mutation or deletion of MKRN3 leads to the initiation of approximately 30% familial CPP [33]. A previous animal experiment concludes that genetic ablation of MKRN3 accelerates the onset of puberty in mice, and the MKRN3/MBD3 axis controls the epigenetic switch of puberty onset in mammals [13], which inspired us to measure MBD3 expression in human sera and to investigate the differential expression pattern of MBD3 in CPP patients and healthy pre-pubertal children and its clinical significance. However, this study was flawed due to the lack of reliable information to ascertain whether MBD3 expression is elevated as a result of increased stimulation of the HPG axis or the arrival of puberty. Thus, it is imperative to conduct animal experiments in the future to obtain a definite conclusion *in vivo*.

## References

[1] Soriano-Guillen L, Argente J. Central precocious puberty, functional and tumor-related. Best Pract Res Clin Endocrinol Metab. 2019;33(3):101262. 10.1016/j.beem.2019.01.003 30733078

[2] Canton APM, Seraphim CE, Brito VN, Latronico AC. Pioneering studies on monogenic central precocious puberty. Arch Endocrinol Metab. 2019;63(4):438–44. 10.20945/2359-3997000000164 PMC10528652 31460623

[3] Sultan C, Gaspari L, Maimoun L, Kalfa N, Paris F. Disorders of puberty. Best Pract Res Clin Obstet Gynaecol. 2018;48:62–89. 10.1016/j.bpobgyn.2017.11.004 29422239

[4] Latronico AC, Brito VN, Carel JC. Causes, diagnosis, and treatment of central precocious puberty. Lancet Diabetes Endocrinol. 2016;4(3):265–74. 10.1016/S2213-8587(15)00380-0 26852255

[5] Calcaterra V, Klersy C, Vinci F, Regalbuto C, Dobbiani G, Montalbano C, et al. Rapid progressive central precocious puberty: diagnostic and predictive value of basal sex hormone levels and pelvic ultrasound. J Pediatr Endocrinol Metab. 2020;33(6):785–91. 10.1515/jpem-2019-0577 32441670

[6] Roberts SA, Kaiser UB. Genetics in endocrinology: genetic etiologies of central precocious puberty and the role of imprinted genes. Eur J Endocrinol. 2020;183(4):R107–17. 10.1530/EJE-20-0103 PMC7682746 32698138

[7] Chen M, Eugster EA. Central precocious puberty: update on diagnosis and treatment. Paediatr Drugs. 2015;17(4):273–81. 10.1007/s40272-015-0130-8 PMC5870137 25911294

[8] Yu HK, Liu X, Chen JK, Wang S, Quan XY. Pelvic ultrasound in diagnosing and evaluating the efficacy of gonadotropin-releasing hormone agonist therapy in girls with idiopathic central precocious puberty. Front Pharmacol. 2019;10:104. 10.3389/fphar.2019.00104 PMC6378315 30804790

[9] Abaci A, Catli G, Anik A, Kume T, Calan OG, Dundar BN, et al. Significance of serum neurokinin B and kisspeptin levels in the differential diagnosis of premature thelarche and idiopathic central precocious puberty. Peptides. 2015;64:29–33. 10.1016/j.peptides.2014.12.011 25572302

[10] Jeong HR, Lee HJ, Shim YS, Kang MJ, Yang S, Hwang IT. Serum Makorin ring finger protein 3 values for predicting central precocious puberty in girls. Gynecol Endocrinol. 2019;35(8):732–6. 10.1080/09513590.2019.1576615 30806524

[11] Chen T, Wu H, Xie R, Wang F, Chen X, Sun H, et al. Serum anti-Mullerian hormone and Inhibin B as potential markers for progressive central precocious puberty in girls. J Pediatr Adolesc Gynecol. 2017;30(3):362–6. 10.1016/j.jpag.2017.01.010 28161677

[12] Li C, Han T, Li Q, Zhang M, Guo R, Yang Y, et al. MKRN3-mediated ubiquitination of poly(A)-binding proteins modulates the stability and translation of GNRH1 mRNA in mammalian puberty. Nucleic Acids Res. 2021;49(7):3796–813. 10.1093/nar/gkab155 PMC8053111 33744966

[13] Li C, Lu W, Yang L, Li Z, Zhou X, Guo R, et al. MKRN3 regulates the epigenetic switch of mammalian puberty via ubiquitination of MBD3. Natl Sci Rev. 2020;7(3):671–85. 10.1093/nsr/nwaa023 PMC8288866 34692086

[14] Marshall WA, Tanner JM. Variations in pattern of pubertal changes in girls. Arch Dis Child. 1969;44(235):291–303. 10.1136/adc.44.235.291 PMC2020314 5785179

[15] Tekin A, Cesur, Aydin K. Comparative determination of skeletal maturity by hand-wrist radiograph, cephalometric radiograph and cone beam computed tomography. Oral Radiol. 2020;36(4):327–36. 10.1007/s11282-019-00408-y 31482463

[16] Xue J, Song W, Si M, Sun C, Li K, Wang W, et al. Serum Kisspeptin and AMH levels are good references for precocious puberty progression. Int J Endocrinol. 2020;2020:3126309. 10.1155/2020/3126309 PMC7700058 33293954

[17] Neely EK, Hintz RL, Wilson DM, Lee PA, Gautier T, Argente J, et al. Normal ranges for immunochemiluminometric gonadotropin assays. J Pediatr. 1995;127(1):40–6. 10.1016/s0022-3476(95)70254-7 7608809

[18] Neely EK, Wilson DM, Lee PA, Stene M, Hintz RL. Spontaneous serum gonadotropin concentrations in the evaluation of precocious puberty. J Pediatr. 1995;127(1):47–52. 10.1016/s0022-3476(95)70255-5 7608810

[19] Livak KJ, Schmittgen TD. Analysis of relative gene expression data using real-time quantitative PCR and the 2(-Delta Delta C(T)) method. Methods. 2001;25(4):402–8. 10.1006/meth.2001.1262 11846609

[20] Maione L, Bouvattier C, Kaiser UB. Central precocious puberty: recent advances in understanding the aetiology and in the clinical approach. Clin Endocrinol (Oxf). 2021;95(4):542–55. 10.1111/cen.14475 PMC8586890 33797780

[21] Vargas Trujillo M, Dragnic S, Aldridge P, Klein KO. Importance of individualizing treatment decisions in girls with central precocious puberty when initiating treatment after age 7 years or continuing beyond a chronological age of 10 years or a bone age of 12 years. J Pediatr Endocrinol Metab. 2021;34(6):733–9. 10.1515/jpem-2021-0114 33856747

[22] Yeh SN, Ting WH, Huang CY, Huang SK, Lee YC, Chua WK, et al. Diagnostic evaluation of central precocious puberty in girls. Pediatr Neonatol. 2021;62(2):187–94. 10.1016/j.pedneo.2020.12.001 33388255

[23] Klein DA, Emerick JE, Sylvester JE, Vogt KS. Disorders of puberty: an approach to diagnosis and management. Am Fam Physician. 2017;96(9):590–9. 29094880

[24] Xu YQ, Li GM, Li Y. Advanced bone age as an indicator facilitates the diagnosis of precocious puberty. J Pediatr (Rio J). 2018;94(1):69–75. 10.1016/j.jped.2017.03.010 28866322

[25] Partsch CJ, Sippell WG. Pathogenesis and epidemiology of precocious puberty. Effects of exogenous oestrogens. Hum Reprod Update. 2001;7(3):292–302. 10.1093/humupd/7.3.292 11392376

[26] Pasternak Y, Friger M, Loewenthal N, Haim A, Hershkovitz E. The utility of basal serum LH in prediction of central precocious puberty in girls. Eur J Endocrinol. 2012;166(2):295–9. 10.1530/EJE-11-0720 22084156

[27] Sathasivam A, Rosenberg HK, Shapiro S, Wang H, Rapaport R. Pelvic ultrasonography in the evaluation of central precocious puberty: comparison with leuprolide stimulation test. J Pediatr. 2011;159(3):490–5. 10.1016/j.jpeds.2011.02.032 21489559

[28] Calcaterra V, De Filippo G, Albertini R, Rendina D, Messini B, Monti CM, et al. Effectiveness of basal LH in monitoring central precocious puberty treatment in girls. J Pediatr Endocrinol Metab. 2021;34(1):45–50. 10.1515/jpem-2020-0386 33189082

[29] Dura-Trave T, Gallinas-Victoriano F, Malumbres-Chacon M, Ahmed-Mohamed L, Guindulain MJC, Berrade-Zubiri S. Clinical data and basal gonadotropins in the diagnosis of central precocious puberty in girls. Endocr Connect. 2021;10(2):164–70. 10.1530/EC-20-0651 PMC7983482 33416514

[30] Fu JF, Liang JF, Zhou XL, Prasad HC, Jin JH, Dong GP, et al. Impact of BMI on gonadorelin-stimulated LH peak in premenarcheal girls with idiopathic central precocious puberty. Obesity (Silver Spring). 2015;23(3):637–43. 10.1002/oby.21010 25645648

[31] Ding Y, Li J, Yu Y, Yang P, Li H, Shen Y, et al. Evaluation of basal sex hormone levels for activation of the hypothalamic–pituitary–gonadal axis. J Pediatr Endocrinol Metab. 2018;31(3):323–9. 10.1515/jpem-2017-0124 29466239

[32] King LR, Siegel MJ, Solomon AL. Usefulness of ovarian volume and cysts in female isosexual precocious puberty. J Ultrasound Med. 1993;12(10):577–81. 10.7863/jum.1993.12.10.577 8246336

[33] Abreu AP, Dauber A, Macedo DB, Noel SD, Brito VN, Gill JC, et al. Central precocious puberty caused by mutations in the imprinted gene MKRN3. N Engl J Med. 2013;368(26):2467–75. 10.1056/NEJMoa1302160 PMC3808195 23738509

